# mGem: Extracellular vesicles in *Leishmania*—secret messengers driving infection and disease

**DOI:** 10.1128/mbio.03239-24

**Published:** 2025-09-22

**Authors:** Patricia Xander, Camila I. de Oliveira

**Affiliations:** 1 Departamento de Ciências Farmacêuticas, Instituto de Ciências Ambientais Químicas e Farmacêuticas, UNIFESP 734878, Diadema, Brazil; 2 Instituto Gonçalo Muniz, FIOCRUZ 37903, Salvador, Bahia, Brazil; 3 Instituto Nacional de Ciência e Tecnologia em Doenças Tropicais (INCT-DT) 453688, Salvador, Bahia, Brazil; Instituto Carlos Chagas, Curitiba, Brazil

**Keywords:** leishmaniasis, immune modulation, pathogenesis, vesicles

## Abstract

Leishmaniasis is a disease caused by *Leishmania* parasites, transmitted by insects, that occurs worldwide. The parasite and parasite-infected cells release extracellular vesicles (EVs), which are involved in numerous biological processes. EVs secreted by *Leishmania* modulate the host cell and, in turn, the immune response. In this review, we focused on two particular EV-related topics: (i) EVs as carriers of *Leishmania* virulence factors and implications in parasite biology, and (ii) the effects of *Leishmania*-derived EVs on the host’s immune response.

## PERSPECTIVE

Leishmaniasis is an insect-transmitted parasitic disease caused by the protozoan *Leishmania,* with worldwide occurrence. Infection by *Leishmania* parasites leads to a broad range of clinical manifestations, which can be grouped into two primary forms: (i) tegumentary leishmaniasis, in which parasites cause ulcers in the skin or mucosal areas, and (ii) visceral leishmaniasis, in which parasites mainly affect the liver, spleen, and bone marrow (1). *Leishmania* parasites have a complex life cycle, alternating between the promastigote life form, present in the invertebrate insect host, and the amastigote. The amastigote is found in the vertebrate host, within professional phagocytes, and is the disease-causing life form. Similar to other eukaryotic organisms, *Leishmania* parasites produce extracellular vesicles (EVs), which mediate cell-to-cell communication, but in the case of leishmaniasis, are very much involved in the host–pathogen interaction, with significant downstream effects regarding pathogenesis. In this review, we focused on two particular EV-related topics: (i) EVs as carriers of *Leishmania* virulence factors and implications in parasite biology and (ii) the effects of *Leishmania*-derived EVs on the host’s immune response.

### EVs as carriers of *Leishmania* virulence factors

To escape this immune response, survive, and propagate, *Leishmania* parasites have developed different strategies (2, 3), and among these is the production of EVs. EVs are particles released by prokaryotic and eukaryotic cells composed of a lipid bilayer that do not replicate and can carry several biological and functional molecules, such as proteins, DNA, RNA, and metabolites (4). Although EVs are highly heterogeneous in terms of size and cargo, they can be classified based on their biogenesis into three categories: exosomes, originating from the endolysosomal pathway; ectosomes or microvesicles, released directly from the plasma membrane; and apoptotic bodies, produced by apoptotic cells (5). Early on, it became clear that EVs secreted by *Leishmania* parasites and *Leishmania*-infected cells play a role in host–pathogen interactions, contributing to pathogenesis (6–9).

Parasites in culture export *Leishmania* virulence factors, and initial mass spectrometry studies found that protein secretion in *Leishmania* also occurs by producing EVs (10). Soon after, exosome secretion was evidenced as a general mechanism of protein delivery to host cells, and *Leishmania*-derived EVs were found within the cytosol of infected macrophages (6). Indeed, macrophage incubation with *Leishmania*-derived exosomes modulated cytokine production and the host’s immune response. Although functional studies have not yet fully characterized EV biogenesis in *Leishmania*, proteomic profiling of *Leishmania*-derived EVs identified proteins involved in the endosomal machinery and the endosomal sorting complex required for transport (ESCRT) pathway (11), which functions in exosome biogenesis and release (12).

Virulence factors, cellular metabolism proteins, and immune mediators have all been described within *Leishmania*-derived EVs (7, 13). Among the virulence factors detected within *Leishmania*-derived EVs, we highlight gp63 (8), the most abundant protein on the parasite’s surface (14), which mediates evasion from complement-mediated lysis (15), degrades proteins from the extracellular matrix (16), and inhibits the p38-MAP kinase pathway in host cells (17). Gp63-containing EVs are also detected in the insect vector (9) and are co-egested with the parasite during parasite transmission to the vertebrate host. Indeed, co-inoculation of *Leishmania* with parasite-derived EVs exacerbated disease, highlighting that the interactions observed at the initial stages of infection go beyond the parasite’s presence or vector components, for example. While several studies characterized EVs produced by *in vitro*-cultured promastigotes (9, 18–21), those investigating EVs within the sand fly are scarce (9). Therefore, it remains to be determined whether the EV cargo found *in vitro* is also found in nature, within infected sand flies. Furthermore, it is unknown whether EVs participate in metacyclic differentiation inside the sand fly vector or if they also interact with the vector’s microbiota.

Besides protein cargo, *Leishmania* RNA virus 1 (LRV1) has been detected in *Leishmania*-derived EVs, showing that EVs can transmit and spread these entities to other parasite populations (22). In this case, EVs act as viral envelopes, facilitating the transmission of LRV1 to the vertebrate host. Significantly, the presence of LRV1 exacerbates leishmaniasis (23), and patients infected with LRV1-harboring *Leishmania* are prone to treatment failure (24). Through EVs, *Leishmania* can exchange virulence factors, as discussed above, and viral entities, as well as drug-resistance genes, using EV secretion (25). Sequencing indicated that EV cargo was derived from non-coding RNA, such as rRNA and tRNA. It will be interesting to determine whether these exchanged small RNAs can, for example, modulate cell signaling or regulate gene expression.

### Effects of *Leishmania*-derived EVs on immune cells

**Fig 1:**
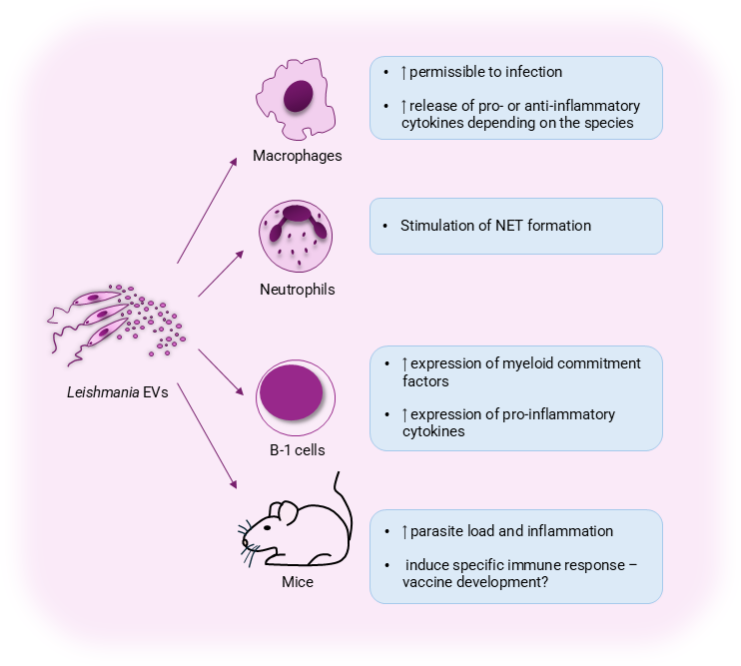
EVs in the context of leishmaniasis. Effects of EVs derived from *Leishmania* parasites on immune cell activation and disease development.

As mentioned before, the amastigote stage is the disease-causing life form of *Leishmania*. Amastigotes reside and multiply within macrophages; therefore, the field has largely explored how infected macrophages respond to *Leishmania*-derived EVs or EVs produced by infected cells. Macrophages exposed to *Leishmania*-derived EVs upregulate the production of pro-inflammatory mediators (26), promote disease progression (20), and engage toll-like receptors (TLRs) to induce immune modulation (27), including M2 polarization (28), which is beneficial to *Leishmania* survival. Mechanistically, *Leishmania*-derived EVs are enriched with polyamines, which are efficiently captured by macrophages, leading to higher infection rates and increased expression of Arginase 1, a known M2 marker (29). Again, gp-63-containing EVs were associated with immune modulation (30), besides their reported disease-exacerbation effect (9). *Leishmania*-derived EVs also promote angiogenesis through stimulation of interleukin-8 (IL-8), granulocyte colony-stimulation factor (G-CSF), and vascular endothelial growth factor-A (VEGF-A) (31), which may have significant effects *in vivo*, especially in the formation of cutaneous ulcers, the main clinical form of tegumentary leishmaniasis. In Fig. 1, we summarize the impact of *Leishmania*-derived EVs and their implications in pathogenesis (Fig. 1).

Most of these studies cited above have been conducted in mouse models or murine cell lines. Still, similar effects have been observed in human cells: pre-stimulation of human macrophages with *Leishmania* EVs induced the production of inflammatory cytokines and inflammasome activation (32). Interestingly, when EV-stimulated macrophages were infected with *Leishmania*, the authors did not observe significant changes in parasite load, suggesting that exposure to EVs modulates innate responses but does not necessarily promote parasite replication. Similarly, human neutrophils exposed to *Leishmania*-derived EVs secrete extracellular traps (NETs) through TLR engagement (33). These NETs, in turn, exerted leishmanicidal effects against promastigotes. Although the two experimental settings are distinct, they show that *Leishmania*-derived EVs activate the human innate response, which, in turn, may have significant effects in shaping the adaptive immune response. Further studies shall address the effects of EVs on other cells, once the parasite is safely established within the definitive host cell (macrophage). Do these bystander cells become more susceptible to infection? Does this contribute to parasite persistence? Do these EVs further stimulate the inflammatory response characteristic of cutaneous leishmaniasis, for example?

Besides macrophages and neutrophils, *Leishmania*-derived EVs modulate B cells, especially B-1 cells—a subtype of B lymphocytes with regulatory properties in immunity. B-1 cells stimulated with *L. amazonensis* EVs showed an increased expression of myeloid commitment factors (34) and tumor necrosis factor-α (TNF-α) (20, 34). These findings suggest a shift of B-1 cells toward a phagocyte-like phenotype, potentially enhancing their microbicidal capacity. On the other hand, mice previously exposed to EVs released by B-1 cells displayed an improved outcome in experimental infection (35). These results raise important questions regarding the dual role of EVs in immunity. While parasite-derived EVs may modulate the host’s immune response to favor infection, host-derived EVs could act as mediators of protective immunity. Understanding the delicate balance between these opposing effects is essential, as it may reveal novel immunomodulatory mechanisms and potential therapeutic strategies against *Leishmania* infection.

### Future directions

Besides the questions we raised along this review, other areas are beginning to be explored such as the use of *Leishmania*-derived EVs for the development of serological tests for leishmaniasis (36). Results show that EVs are recognized by sera from infected individuals with high (95%) sensitivity and specificity (100%). Analysis of sera from infected dogs showed the presence of EVs carrying microRNAs (37) and suggests the possibility of employing EVs as biomarkers of disease, for example. The therapeutic use of EVs is also being pursued: inoculation of EVs derived from mesenchymal stem cells (MSCs) partially controlled lesion development in experiment infection without altering the parasite load. Use of MSC-derived EVs in association with chemotherapy, however, showed an additive effect (38). Thus, EVs sourced from MSCs or B1 are poised to exert immunomodulatory properties and, thus, can be explored as host-directed therapies, especially in clinical forms of leishmaniasis that are highly inflammatory.
